# Revision der anatomischen Schulterprothese

**DOI:** 10.1007/s00132-022-04337-3

**Published:** 2023-01-19

**Authors:** Frieder Mauch, Jochen Huth

**Affiliations:** Departement für Obere Extremität/MRT, Sportklinik Stuttgart, Taubenheimstr. 8, 70327 Stuttgart, Deutschland

**Keywords:** Arthroskopie, Infekt, Prothesenlockerung, Reoperation, Schultertotalersatz, Arthroscopy, Infection, Prosthesis loosening, Reoperation, Total shoulder replacement

## Abstract

Mit der steigenden Anzahl der Primärprothetik nehmen die Revisionen der anatomischen Prothese einen immer höheren Stellenwert ein. Die häufigsten Revisionsgründe sind Glenoidlockerung, einschließlich Protrusion, Insuffizienz der Rotatorenmanschette, einschließlich Instabilität, und Früh‑/Spätinfekt. Der glenoidale Defektaufbau kann mit einem Autograft oder Allograft erfolgen. Er wird je nach Größe und Situation ein- oder zweizeitig durchgeführt. Die in den letzten Jahren immer häufiger eingesetzten metaphysär verankerten Prothesen und Kurzschaftprothesen haben die humerale Revision deutlich vereinfacht. Einen anderen Weg beschreiten die Plattformsysteme mit der Möglichkeit der Konversion ohne höhergradige Eingriffe am Glenoid oder Wechsel des Schaftes. Intraoperative Komplikationen treten vor allem humeral auf. Postoperative Komplikationen sind neben der Luxation die Komponentenlockerung und der Infekt. Der Wechsel einer anatomischen Prothese auf eine inverse Prothese zeigt bessere klinische Ergebnisse, sowie niedrigere Komplikationsraten als der Wechsel auf nochmals eine anatomische Prothese.

## Abstract (en)

With the increasing number of primary arthroplasties, revisions of anatomical prostheses are becoming increasingly important. The most common reasons for revision are glenoid loosening, including protrusion, rotator cuff insufficiency, including instability, and early/late infection. The reconstruction of glenoid defects can be done with an autograft or allograft. Depending on the size and situation, it is carried out in one or two stages. The stemless humeral head replacement and the short-stem prostheses that have been used more frequently in recent years have significantly simplified humeral revision. Platform systems take a different approach with the option of conversion without major interventions on the glenoid or revision stem. Intraoperative complications mainly occur on the humeral side. Postoperative complications include dislocation, component loosening, and infection. Revision of anatomical to reverse arthroplasty shows better clinical outcomes and lower complication rates than anatomical revision.

Im Laufe der letzten Jahre nahm die Anzahl implantierter Schulterprothesen kontinuierlich zu. Gleichzeit steigt in den Schulterzentren die Zahl an Revisionsoperationen. Dieser Trend wird sich die nächsten Jahre fortsetzen. Der Artikel gibt einen Überblick über die Gründe für Revisionen und präsentiert Lösungsmöglichkeiten bei verschiedenen Ursachen.

## Einleitung

Die Schulterprothetik hat in den letzten Jahren einen ständigen Zuwachs erfahren. Neben der Entwicklung in der inversen Prothetik steht die Weiterentwicklung der anatomischen Prothetik, insbesondere in den letzten Jahren auf der glenoidalen Seite. Unterschiedliche Glenoiddesigns versuchen, die glenoidale Problematik zu lösen. Mit der steigenden Anzahl der Primärprothetik nehmen die Revisionen schon heute an einem Schulterzentrum einen großen Platz ein. Es ist davon auszugehen, dass in Zukunft die Revision der anatomischen Prothese einen noch größeren Stellenwert erlangen wird [13]. Dieser Artikel soll Ihnen einen Überblick geben über:

- Die Gründe für die Revisionen.
- Lösungsmöglichkeiten für die glenoidale Lockerung.
- Lösungsmöglichkeiten für die humerale Seite einschließlich Plattformsysteme.
- Lösungsmöglichkeiten für die Rotatorenmanschetteninsuffizienz, einschließlich Dislokation/Instabilität.
- Lösungsmöglichkeiten ohne Prothesenwechsel (einschließlich Arthroskopie).
- Ergebnisse der Revision bei anatomischer Prothese.

## Revisionsgründe

Die Endoprothesenregister werden wegen ihrer Fallzahlen gerne herangezogen, um die Revisionsgründe nach Schulterendoprothesenoperationen zu erfahren. Die Registerdaten spiegeln meist die Versorgungsrealität wider, da sie, im Gegensatz zu klinischen Studien, keine strikten Ein- und Ausschlusskriterien benutzen. Die Überlebensrate der Prothese wird in allen Registern als primärer Outcome-Parameter verwendet. Die Erfassung auf der herstellerspezifischen Ebene lässt einen unabhängigen Vergleich der im Register eingegeben Prothesenmodelle zu.

Die häufigsten Gründe für die Revision einer anatomischen Schulterprothese sind somit:

- Glenoidlockerung, einschließlich Protrusion
- Insuffizienz der Rotatorenmanschette, einschließlich Dislokation/Instabilität
- Bewegungseinschränkungen und Schmerzen
- Früh- oder Spätinfekt
- Lockerung der humeralen Komponente
- Implantatversagen
- periprothetische Fraktur

Sie können einzeln oder in Kombination auftreten [2, 4, 22].

Allgemeine Hinweise zur Revision:

- Bei Schmerzen immer an Infekt und/oder Instabilität denken.
- Genaue Analyse des Röntgenbildes/CT:
  - Hochstand/Luxation?
  - Nicht korrekte humerale oder glenoidale Version?
  - Lysezeichen?
- Ultraschalldiagnostik eignet sich sehr gut für die Darstellung der Rotatorenmanschette.
- Blutentnahme mit CRP, Leukozytenbestimmung und Differenzialblutbild.
- Bei Glenoidlockerung und Schmerzen nicht zu lange mit der Revision warten.
- Entnahme von Proben für die Histologie, Mikrobiologie, sowie Abstrichentnahme.
- Jetlavage bei jeder Revisionsoperation.
- Revisionssets und Langschäfte müssen vorhanden sein.

## Aseptische oder septische Glenoidlockerung, einschließlich Protrusion

Die Glenoidlockerung stellt den häufigsten Grund für die Revision dar [15]. Zum Vorgehen in der septischen Situation wird auf den Artikel von Hudek et al. in dieser Ausgabe verwiesen. Bei der Bildgebung ist neben dem Röntgenbild immer eine CT-Diagnostik mit Metallartefaktunterdrückung erforderlich. Eine 3‑D-Rekonstruktion mit Spiegelung der Gegenseite kann für die Volumenbestimmung des Defektes hilfreich sein. Die knöcherne Glenoidrekonstruktion wird in dieser Ausgabe in einem eigenen Beitrag von Herrn Seebauer behandelt. Hier werden nur die grundsätzlichen Punkte für die Revision der anatomischen Prothese beleuchtet.

Die glenoidale Defekteinteilung erfolgt meistens anhand der Einteilung nach Antuna et al. in periphere, zentrale und kombinierte Defekte. Sie wurde von Gohlke et al. modifiziert und stellt im klinischen Alltag den Standard dar [1, 16]. Der Aufbau des Glenoids kann mit einem Autograft oder Allograft erfolgen. Er wird je nach Größe und Situation ein- oder zweizeitig durchgeführt.

Eine einzeitige Revision ist meistens bei zentralen Defekten (erst- und zweitgradige Defekte) möglich. Hier erfolgt die Auffüllung des Defektes meist durch ein sogenanntes „impaction grafting“ mit anschließender Modellierung und Fixierung durch ein „metal back“ aus der inversen Prothetik. Der zentrale Zapfen und divergierende winkelstabile Schrauben ermöglichen die zementfreie Rekonstruktion von Glenoiddefekten und verbessern die Einheilung des Glenoidaufbaus [5]. Eine erneute Zementierung eines Polyethylenglenoids auf ein „impaction grafting“ ist im Bereich der Schulter nicht möglich [7, 19].

Bei exzentrischen/peripheren Defekten (Grad 3) erfolgt die Defektfüllung je nach Operateur und Situation ein- oder zweizeitig. Oberstes Ziel ist die neben dem Debridement die korrekte Wiederherstellung der Anatomie mit Lateralisierung und der sicheren zentralen Zapfen‑/Schraubenpositionierung nach dem 3‑Säulen-Prinzip mit zentraler Abstützung durch den Zapfen und Flächenpressung durch die winkelstabilen Schrauben [17]. Hierbei wird der Defekt zunächst mit Hochgeschwindigkeitsfräsen so präpariert, dass das Knochentransplantat eingepasst werden kann. Die Fixierung über die Platte erfolgt mit sekundärer Winkelstabilität, sodass hier eine Kompression auf den eingebrachten Span erfolgt, bevor die eigentliche Winkelstabilität erreicht wird [30]. Unterschiedliche Peglängen erlauben die Positionierung im originären Knochen. 10 mm oder ein Drittel der Peglänge scheinen auszureichen, um eine ausreichende Stabilität zu gewährleisten [17, 25].

Bei großen Knochendefekten mit ausgeprägter Medialisierung wird ein zweizeitiges Vorgehen bevorzugt. Hier ist eine sichere zentrale Fixierung meist nicht mehr möglich. Genügend Substanz wird meist durch die Allograftversorgung erreicht.

Eine Alternative stellt in diesen Fällen eine Versorgung mit einem Sonderimplantat mit individueller Verankerungsmöglichkeit dar (Materialise, Leuven, Belgien). Nach durchgeführter CT-Untersuchung, gemäß einem speziellen Protokoll, wird eine 3‑D-Rekonstruktion der Skapula und des Defektes erstellt. Mittels eines „statistical shape models“ wird das native Glenoid virtuell hochgerechnet und rekonstruiert und somit die originäre „joint line“ bestimmt. Das Implantat ist an den Defekt angepasst und nutzt die maximale Kontaktfläche. Nach Absprache mit dem Chirurgen wird dann das Implantat nach Planungsabschluss erstellt. Es stehen zwei Verankerungsmöglichkeiten zur Verfügung, entweder mit Zapfen und nichtwinkelstabilen Schrauben oder ohne Zapfen mit winkelstabilen Schrauben. Die Implantate bestehen aus einer metallischen Basisplatte und einer porösen Oberfläche an der zum Knochen gewandten Seite, die den Knochendefekt ausfüllt (Titanlegierung). Die Herstellungs- und Lieferfrist ab dem Zeitpunkt der Genehmigung durch den Chirurgen beträgt etwa 8–10 Wochen. Die Lieferung besteht aus Planung, Originalimplantat, Skapula- und Implantatproben sowie Bohr- und Positionierungsschablonen.

Im eigenen Vorgehen wird bei „unsicherer“ Situation das zweizeitige Vorgehen bevorzugt. Hierbei wird nach Vorbereitung des Defektes das Allograft unter BV-Kontrolle mit temporären Schrauben fixiert. Der proximale Humerus wird schon für die endgültige Versorgung vorbereitet und mit einem temporären Antibiotikum-Zementspacer versorgt. Es werden Proben für die Histologie, Mikrobiologie sowie Abstriche entnommen (Bebrütung wegen Cutibacterium acnes mindestens 14 Tage). Eine Jetlavage ist bei jeder Revisionsoperation obligat.

**Figure 1:**
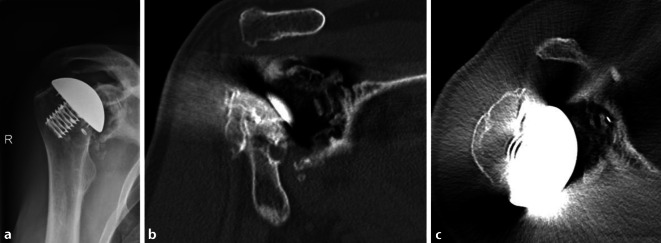

**Figure 2:**
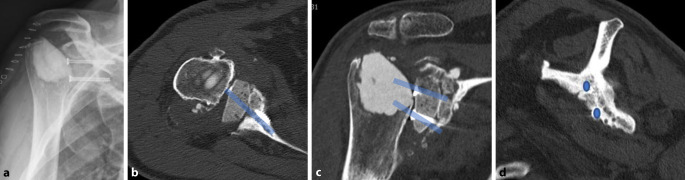

**Figure 3:**
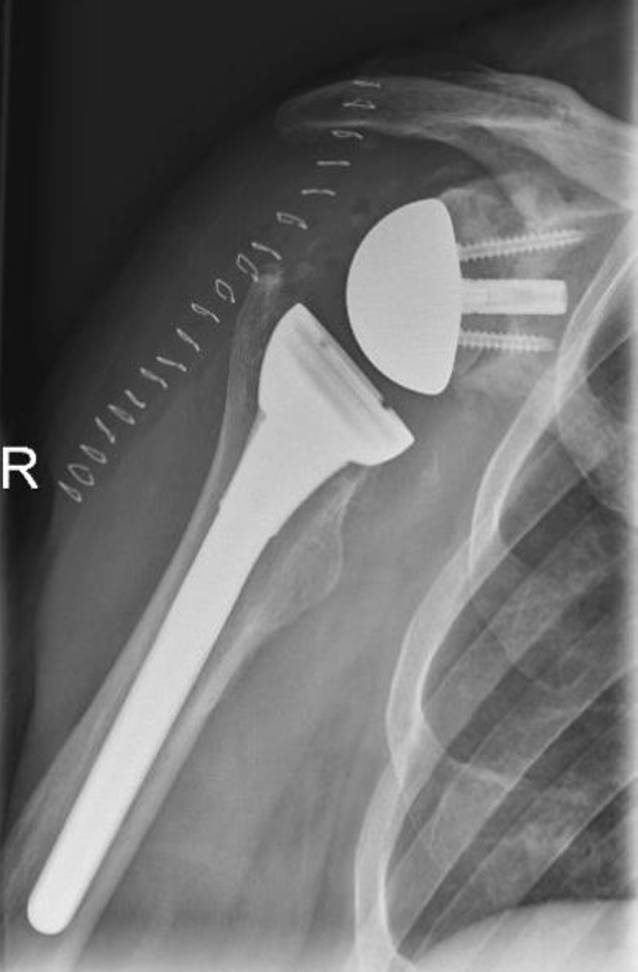

Eine postoperative CT kann für die „Navigation“ des endgültigen zentralen Zapfens und die Schraubenplatzierung benutzt werden (Abb. 1a–c, Abb. 2a–d und Abb. 3).

## Humerale Revision

Über die ausführliche Darstellung der humeralen Knochendefekte wird auf den Beitrag von Herrn Prof. Frank Gohlke verwiesen.

Die in den letzten Jahren immer häufiger eingesetzten metaphysär verankerten Prothesen und Kurzschaftprothesen haben die humerale Revision deutlich vereinfacht. Eine Entfernung und ein Wechsel auf eine Schaftprothese sind fast immer möglich. Einen anderen Weg beschreiten die Plattformsysteme mit der Möglichkeit der Konversion ohne höhergradige Eingriffe am Glenoid oder Wechsel des Schaftes. Auf das Metallback am Glenoid kann im Revisionsfall die Glenosphere fixiert werden kann. Wichtiger scheint aber die Möglichkeit des Umbaus auf der humeralen Seite. Hier kann in der Theorie der Schaft belassen werden. Operationszeit, Komplikationen und Blutverlust können dadurch verringert werden [23].

Darüber hinaus sind Gründe für notwendige Revisionen die Schaftlockerung und periprothetische Frakturen, aber auch Revisionen und die Entfernung des Schaftes aufgrund einer Glenoidlockerung oder Rotatorenmanschetteninsuffizienz sind sehr häufige Wechselgründe. Problematisch sind vor allem zementfreie Titanschäfte, die knöchern sehr fest integriert sind. Ein Ausbau ohne größeren Knochenverlust ist meist nicht möglich. Der Operateur muss die zu revidierende Prothese kennen, oder bei der entsprechenden Firma das notwendige Revisionsinstrumentarium bestellen. Ein Ausschlagversuch mit dem entsprechenden Ausschläger sollte am Anfang immer versucht werden. Mit Klingenmeißeln kann dann die Prothese vorsichtig freigemeißelt werden. Der Ausschlagversuch kann mehrmals wiederholt werden [5]. Bei ausbleibendem Erfolg kann mit einer feinen Säge der Schaft gespalten und vorsichtig aufgedehnt werden. Cerclagen sichern nach Implantation das Ergebnis.

Die Revision des zementierten Schaftes ist meist einfacher mit nachfolgender Zement-in-Zement-Fixation möglich. Ist dies nicht möglich, muss der Zement so weit wie möglich auch durch entsprechende Fähnchenmeißel entfernt werden. Ein nicht vorhandener Zementstopper bei der Erstoperation, sowie Osteoporose können hier die Wechseloperation erheblich erschweren.

## Insuffizienz der Rotatorenmanschette, einschließlich Dislokation/Instabilität

Hier werden superiore Defekte von ventralen Defekten unterschieden. Die Integrität der Rotatorenmanschette, insbesondere der Subskapularissehne ist der Schlüsselfaktor für ein gutes funktionelles Ergebnis nach anatomischer Prothetik. Die superioren Rotatorenmanschettendefekte stellen aber auch über die Jahre einer Standzeit sicher eine Art natürliche Degeneration und Verschleiß mit sukzessiver Migration des Oberarmkopfes nach kranial dar und können insbesondere im Langzeitverlauf nicht immer als Komplikation bezeichnet werden.

In einer Metaanalyse von Levy et al. bei 1338 untersuchten Schultern nach 6,6 Jahren stellte die Insuffizienz der Rotatorenmanschette mit 11,3 % (±7,9 %) eine häufige „Komplikation“ dar [26]. Young et al. fanden in einer Multicenteranalyse nach einem Beobachtungszeit von 8,6 Jahren in 16,8 % der Fälle sekundäre Rotatorenmanschettenschäden. Die Überlebensrate von Schulterprothesen mit intakter Manschette war, ausgehend von 100 % nach 5 Jahren, nach 10 Jahren auf 84 % und nach 15 Jahren auf 45 % abgesunken [33]. In der Arbeitsgruppe um Valenti et al. [21] wurden Patienten mit Instabilität untersucht. Sie konnten zeigten, dass in den meisten Fällen der superioren Instabilität die Rotatorenmanschettenmassenruptur ursächlich war und die isolierte Subskapularissehnenruptur sich bei Patienten mit isolierter vorderer Instabilität zeigte. Die Therapie dieser superioren Defekte besteht bei Schmerzen im Wechsel auf eine inverse Prothese. Bei geringer Beschwerdesymptomatik kann ein abwartendes Verhalten in Erwägung gezogen werden.

Entezari et al. untersuchten in ihrer Arbeit 25 Patienten mit Subskapularissehnenruptur. Ein Rekonstruktionsversuch sollte bei eher jüngeren Patienten, mit weniger Komorbiditäten und akuter Symptomatik in Erwägung gezogen werden. Ältere, kranke und Patienten mit eher chronischer Anamnese kann der Wechsel auf inverse Prothese empfohlen werden [12]. Da die Therapie der Insuffizienz der Rotatorenmanschette sehr häufig in der Wechseloperation endet, muss der Operateur bei der Primäroperation höchste Sorgfalt auf Weichteilmanagement, korrekte Inklination und Version legen. Eine Overstuffing muss vermieden werden.

Eine vorausgehende Rotatorenmanschettenrekonstruktion führte in einer anderen Studie im Falle einer späteren inversen TEP zu schlechteren Scores und höherem Schmerzniveau als ohne Voroperation an der Rotatorenmanschette [31].

## Bewegungseinschränkung und „unklare“ Schmerzen

Mit der arthroskopischen Evaluation besteht die Möglichkeit zur erweiterten Diagnostik, aber auch Therapie. Insbesondere die Inspektion der Weichteile und die Evaluation des Glenoids, sowie Probeentnahme für Diagnostik des Infektes sind einfach möglich. Die Arthroskopie erfolgt in der für den Operateur vertrauten Lagerung. Das dorsale Zugangsportal erfolgt etwas kranialer und lateraler, um Schaden am Glenoid und an der Oberfläche der Prothese zu vermeiden. Die weiteren Portale werden unter Sicht angelegt und folgen dem normalen Standardvorgehen. Eine weitere Besonderheit stellt das „Spiegelphänomen“ dar. Hierbei kommt es zu Spiegelung der Optik und der Instrumente an der Prothesenoberfläche. Das „Wegdrehen“ der 30-Grad-Optik minimiert diesen Effekt.

### Bewegungseinschränkung/Impingement

Patienten mit Bewegungseinschränkungen bzw. unklaren Schmerzen bei normaler Bildgebung stellen neben den oben genannten Patienten ebenfalls ein Problem dar [14]. In der frühen postoperativen Phase werden Physiotherapie und Eigenübungen eingesetzt. Die akute Subskapularissehnenruptur muss ausgeschlossen werden. Bei persistierender Bewegungseinschränkung kann die arthroskopische Evaluation in Betracht gezogen werden. Adhäsionen und Narben im Rotatorenintervall und medialen glenohumeralen Ligament werden gelöst und der Subskapularis vorsichtig mobilisiert. Bei Kontraktionen der posterioren Kapsel wird die Kamera nach ventral umintubiert und die dorsalen Kapselanteile vorsichtig entfernt. Die kaudalen Kapselanteile werden zunächst belassen. Der subakromiale Raum wird inspiziert und die gesamte Bursa mit den Verwachsungen entfernt. Danach erfolgt die Inspektion der Rotatorenmanschette. Bei persistierender Bewegungseinschränkung kann das kaudale Release in Betracht gezogen werden [11, 18].

### Verdacht auf Low-Grade-Infekt

Für die Abstrichentnahme, histologische und mikrobiologische Untersuchung stellt die Arthroskopie ein sehr gutes Tool dar, um ausreichend Material für die Sicherung der Diagnose zu gewinnen. Im eigenen Vorgehen verfolgen wir den Algorithmus von Hudek (siehe Beitrag in dieser Ausgabe). Es werden 5 Proben für die Mikrobiologie und 2 Proben für die Histologie entnommen. Die Bebrütung sollte mindestens 14 Tage erfolgen. Von einem Infekt mit dann notwendigem Wechsel kann bei positivem Befund < 8 Tagen und gleichzeitig positiver Histologie ausgegangen werden. Von keinem Infekt mit Erhaltungsversuch je nach Situation wird bei positivem mikrobiologischem Ergebnis > 11 Tagen und negativer Histologie, sowie negativer Bebrütung bis 14 Tage ausgegangen.

Weitere mögliche Indikationen zur Arthroskopie können die Evaluation der akut aufgetreten Rotatorenmanschettenruptur, die Diagnostik der Instabilität und die Bizepssehnenpathologie sein [28].

## Ergebnisse der Revisionsendoprothetik

Das Verständnis und die Datenlage der Revisionen sind immer noch limitiert. Es sei erwähnt, dass es sich bei dieser Darstellung um eine sehr heterogene Patientengruppe handelt. Meist werden Ergebnisse von retrospektiven Fallstudien mit einer großen Variabilität der klinischen Ergebnisse herangezogen. Patienten, die wegen einer Glenoidlockerung operiert werden, können unterschiedliche Indikationen bei Primäroperation aufweisen, die dann das Ergebnis beeinflussen. Es kann aber auch sein, dass eine nicht diagnostizierte chronische Infektion das Ergebnis aufgrund einer Rotatorenmanschetteninsuffizienz maskiert.

Unbestritten ist, dass wir mehr Information über die klinischen Ergebnisse und Standzeiten der Revisionsendoprothesen benötigen, um die in Zukunft weiter ansteigenden Zahlen wissenschaftlich abzusichern und das richtige Implantat auswählen zu können. Ein wichtiger Schritt ist hier die Aufnahme der Standzeiten und Erfassung der klinischen Ergebnisse der Revisionsprothetik in den Prothesenregistern. Keines der neun nationalen Prothesenregister mit ihren Ergebnissen und Standzeiten für die Primärprothetik, erfasst im Moment das Outcome in der Revisionsendoprothetik [3]. Des Weiteren sollte man die Unterschiede zwischen Nordamerika und Europa in Betracht ziehen. In den USA wurden in der Vergangenheit mehr Hemiprothesen als in Europa primär implantiert und die inverse Prothese hat erst im Jahre 2003 die FDA-Zulassung bekommen. Dies hat auch Auswirkungen auf das Revisionsimplantat. In den europäischen Studien haben die Patienten demnach eine 37 % höhere Wahrscheinlichkeit, die inverse Prothese als Revisionsimplantat zu erhalten [24].

Davies et al. untersuchten in ihrem systematischen Review die Revision von Hemiprothesen und Totalprothesen (TSA) [10]. Insgesamt wurden 15 Arbeiten (11 TSA/4 Hemi) eingeschlossen. Die Revision erfolgte in 7 Studien von TSA auf inverse Schulterprothese und bei 4 Studien von TSA auf TSA. Im Vergleich der beiden Revisionsimplantate zeigten sich bei der Revision auf eine inverse Prothese bessere klinische Ergebnisse.

In einem systematischen Review aus dem Jahr 2021 konnten Ravi et al. insgesamt 112 Studien mit 5379 Schultern einschließen. Der mittlere Follow-up betrug 24 Monate [29]. Häufigste Gründe für die Revision waren Glenoidlockerung, Komponentenlockerung, Infektion, periprothetische Fraktur, Rotatorenmanschetteninsuffizienz und Instabilität. Das mittlere Alter bei Revision betrug 67 Jahre, der Frauenanteil betrug 60 %. Die am häufigsten durchgeführten Indexoperationen waren Hemiprothesen und die Mehrheit der Schultern wurde auf eine inverse Prothese gewechselt (67 %). Intraoperative Komplikationen fanden sich in 50 der 112 Studien (45 %). Von den 2915 Schultern kam es bei 230 Schultern (8 %) zu intraoperativen Komplikationen. Iatrogene Frakturen traten hier mit 70 % bei Weitem am häufigsten auf. In den überwiegenden Fällen handelte es sich um Frakturen am Humerus. Die Entfernung des Schaftes stellt somit den kritischsten Moment in der Revision dar und hat die meisten intraoperativen Komplikationen zur Folge. Modularität, die immer weitere Verbreitung von metaphysär verankerten Prothesen sowie Kurzschaftprothesen werden wahrscheinlich in Zukunft diese Komplikationen reduzieren.

Bei der Gruppe der anatomischen Prothesen zeigte sich eine höhe postoperative Komplikationsrate

Glenoidfrakturen spielen dagegen heute schon keine große Rolle. Nervenverletzungen werden bei 4 % und Gefäßverletzungen mit unter 1 % angegeben. Weitere Komplikationen waren: Zementaustritt (7 %), Schaftperforation (4 %) und unspezifischen Komplikationen mit 9 %. Von den 3843 erfassten Schultern wiesen 825 postoperative Komplikationen auf (21 %). Instabilität war mit 26 % die häufigste postoperative Komplikation. Weitere Komplikationen waren Komponentenlockerung, -dislokation (19 %) und Infektion (16 %) sowie periprothetische Frakturen (12 %). Die Reoperationsrate lag bei 15 %. Bei der Unterscheidung zwischen Wechsel auf anatomische Prothese und inverse Prothese zeigte sich eine höhere postoperative Komplikationsrate in der Gruppe der anatomischen Prothesen.

Klinische Ergebnisse wurden in 55 von 112 Studien erhoben (49 %). 87 % der erfassten Patienten, zeigten eine Verbesserung bezüglich ASES-Score und Constant-Murley-Score. Nur fünf Studien zeigten keine Verbesserung. Patienten, die auf eine inverse Prothese gewechselt wurden, zeigten signifikant bessere klinische Ergebnisse als Patienten, die nochmals auf eine anatomische Prothese gewechselt wurden.

In einer weiteren Metaanalyse untersuchten Bois et al. die klinischen Ergebnisse und Komplikationen der inversen Prothese im Revisionsfall für unterschiedliche Indexoperationen [6]. Insgesamt wurden 43 Studien mit 1041 Schultern inkludiert. 14 Studien untersuchten die Revision der anatomischen Prothese (TSA-Gruppe). Weitere Indexoperationen waren Hemiprothesen, inverse Schulterprothesen, Z. n. Plattenosteosynthese und Z. n. fehlgeschlagener Rotatorenmanschettenrekonstruktion. Der klinische Parameter Schmerz verbesserte sich in allen Indexgruppen, wenn auch nicht signifikant. Die Werte für die Revision der anatomischen Prothese verbesserten sich von 4,4 auf 11,4 im CS, sowie respektive von 20,8 auf 33,0 im ASES-Score. Die Analyse der subjektiven Parameter zeigte, dass die TSA-Gruppe eine signifikante Verbesserung im CS zeigte, währende im ASES-Score die Verbesserungen der subjektiven Befindlichkeit kein Signifikanzniveau erreichte.

Bei der Bestimmung der Beweglichkeit fanden sich alle Bewegungsrichtungen bis auf die Außenrotation der inversen Indexgruppe verbessert. In einem kürzlich durchgeführten systematischen Review stellte sich eine Flexion zwischen 90 und 135° als ausreichend für den täglichen Alltag heraus [27]. Außerdem sind die meisten Tätigkeiten im Alltag unter 90° Elevation auszuführen [8]. Die Revision der anatomischen Prothesengruppe zeigte in allen Bewegungsrichtungen Verbesserungen, auch wenn sie nicht ganz das Signifikanzniveau erreichte. Sie erreichte aber dieses notwendige Bewegungsausmaß.

Die Gesamtkomplikationsrate betrug in der Analyse 22,9 %. In der TSA-Gruppe mit 14 Studien fanden sich 23,6 % Komplikationen. Die häufigste Komplikation mit 5,5 % stellte die Skapulafraktur dar. Weitere Komplikationen waren Luxation/Instabilität (4,5 %), Basisplattenausbruch (3,2 %), aseptische Lockerung (3,2 %) und Protheseninfektion (2,3 %). Die höchste Anzahl mit 56,2 % fanden sich in der Revisionsgruppe der inversen Prothesen. Die Gesamtrevisionsrate betrug 9,0 %, mit der niedrigsten Rate in der TSA-Gruppe mit 7,1 %.

Zusammenfassend zeigt die Revision der anatomischen Prothese auf eine inverse Prothese in dieser Metaanalyse gute Ergebnisse bezüglich Schmerz und Beweglichkeit. Die häufigste Komplikation stellt die Skapulafraktur dar. Die Revisionsrate ist mit 7,1 % unterdurchschnittlich.

In einem systematischen Review von Kirsch et al. wurden unterschiedliche Plattformsysteme hinsichtlich Komplikationen, Operationsdauer und Blutverlust verglichen. 7 Studien mit 296 Schultern wurden inkludiert. Intraoperative Komplikationen, Reoperationen, Operationsdauer sowie Blutverlust waren bei belassenem Schaft niedriger als bei Schaftwechsel [23]. Bezüglich des klinischen Outcomes konnten Crosby et al. zeigen, dass die klinischen Parameter bei belassenem Schaft etwas besser waren als im Falle eines Schaftwechsels [9]. Allerdings ist der Einbau eines konvertierbaren Systems keine Garantie die Wechseloperation ohne Entfernung der Komponenten durchführen zu können. Die nicht optimale Positionierung bei der Indexoperation, sowie kontrakte Weichteile sind die häufigsten Gründe für einen Schaftwechsel [20, 32].

Zusammenfassend zeigen die Metaanalysen, dass der Wechsel einer anatomischen Prothese auf eine inverse Prothese bessere klinische Ergebnisse, sowie niedrigere Komplikationsraten aufweisen als der Wechsel auf nochmals eine anatomische Prothese. Die häufigsten intraoperativen Komplikationen betreffen noch den Humerus. Postoperativ stehen vor allem Instabilität und Skapulafrakturen im Mittelpunkt. Wissen über Standzeiten in der Revisionsprothetik gibt es wenig. Die Plattformsysteme mit Modularität reduzieren die Komplikationen und Reoperationen, wenn der Schaft belassen werden kann.

## Fazit für die Praxis

- Mit der steigenden Anzahl der Primärprothetik nehmen die Revisionen der anatomischen Prothese einen immer höheren Stellenwert ein.
- Die Glenoidlockerung und die Rotatorenmanschetteninsuffizienz sind die häufigsten Gründe für eine Revision.
- Bei Schmerzen immer an Infekt und/oder Instabilität denken.
- Glenoidale Defekte werden je nach Situation ein- oder zweitzeitig versorgt.
- Intraoperative Komplikationen treten vor allem humeral auf.
- Postoperative Komplikationen sind neben der Luxation die Komponentenlockerung und der Infekt.
- Der Wechsel einer anatomischen Prothese auf eine inverse Prothese zeigt bessere klinische Ergebnisse, sowie niedrigere Komplikationsraten, als der Wechsel auf nochmals eine anatomische Prothese.

## Abkürzungen

ASES: American Shoulder and Elbow Surgeons
BV: Bildverstärker
CRP: C‑reaktives Protein
CS: Constant-Murley-Score
FDA: Food and Drug Administration
TSA: Totalprothesen
